# Detecting latent safety threats in an interprofessional training that combines in situ simulation with task training in an emergency department

**DOI:** 10.1186/s41077-018-0083-4

**Published:** 2018-11-23

**Authors:** Thomaz Bittencourt Couto, Joyce Kelly Silva Barreto, Francielly Cesco Marcon, Ana Carolina Cintra Nunes Mafra, Tarso Augusto Duenhas Accorsi

**Affiliations:** 1 Hospital Israelita Albert Einstein, Av. Albert Einstein, 627/701, Bloco A–1 subsolo, Sao Paulo, SP CEP 05651-901 Brazil; 2 Centro de Simulação Realística Albert Einstein, Sao Paulo, Brazil; 3 Instituto Israelita de Ensino e Pesquisa Albert Einstein, Sao Paulo, Brazil

**Keywords:** Simulation, In situ simulation, Task training, Interprofessional, Emergency medicine, Pediatric emergency medicine

## Abstract

**Background:**

During in situ simulation, interprofessional care teams practice in an area where clinical care occurs. This study aimed to detect latent safety threats (LST) in a training program, which combined in situ simulation scenarios with just-in-time and just-in-place self-directed task training in an emergency department. We hypothesized this simulation-based training in actual care areas allows the detection of at least one LST per simulation scenario.

**Methods:**

This prospective observational study (April 2015–March 2016) involved 135 physicians, nurses, and nurse technicians. Training themes selected were arrhythmia, respiratory insufficiency, shock, and cardiopulmonary resuscitation. Simulation weeks occurred every 3 months, with three 10-min scheduled in situ simulation scenarios alternating for each theme daily. The scenarios were followed by co-debriefing by two facilitators (a physician and a nurse). LST were identified by facilitators using a debriefing checklist. Additionally, a room was set up with task-trainers related to each theme.

**Results:**

The number participants in scenarios was 114 (84% of the population) and in task-training, 101. The number of scenario cancelations was nine, making the final total number to 49 of 58 proposed. Fifty-six LST were observed, with an average of 1.1 per scenario. LST were divided into four categories: equipment (*n* = 23, 41.1%), teamwork (*n* = 12, 21.4%), medication (*n* = 11, 19.6%), and others (*n* = 10, 17.9%). There was a higher proportion in equipment-related LST (*p* < 0.01).

**Conclusions:**

The training allowed a high rate of detecting LST regardless of theme. Equipment-related LST were more frequently found.

**Electronic supplementary material:**

The online version of this article (10.1186/s41077-018-0083-4) contains supplementary material, which is available to authorized users.

## Background

The most likely place for serious safety events to occur is in hospitals’ emergency departments. To minimize such occurrences, simulation-based training of emergency care teams is valuable [1]. Many factors, such as high uncertainty, high cognitive load, high decision density, multiple interruptions, and a narrow window for decision-making, are inherent to the emergency department and may affect care. Team performance is affected by modifiable factors, such as teamwork quality, communication, overcrowding, environmental noise, inadequate supervision, lack of feedback, fatigue, sleep deprivation, multiple handovers of care, and even the department architecture [2].

During in situ simulation, professionals practice in an area where clinical care occurs [3]. Instead of training in a simulation center with incomplete teams and professionals that will not necessarily work together, in in situ simulation, the actual interprofessional care teams interact in their own environment. This simulation modality enables assessing system competence and detecting latent safety threats (LST), or the conditions that may risk patient safety [4].

Patterson et al. (2013) assessed the impact of simulation-based training on pediatric emergency department staff conducted in a simulation center; they found changes in safety attitudes of interprofessional team and significant reduction in serious safety events [5]. In two different follow-up studies by the same group of researchers, the findings were as follows: a 0.8 LST was found for each in situ simulation performed [4]; emergency department providers’ preferred in situ training over training in a simulation center; and high quality teamwork among professionals was developed because of frequent simulation training [6].

In procedural simulation, a task trainer is used to assist in the process of learning to complete a technical skill or a procedure. In just-in-place task training, skills are practiced in an area they are most likely needed, whereas in just-in-time task training, skills are practiced at moments close to the need of using them [7]. Both simulation-based techniques allow deliberate practice to prepare for low frequency, high acuity events. To practice in this manner likely allows higher skill retention and is a rational use of simulation resources, delivering training for those in most need of it [8–10].

We designed a hybrid training program after a serious safety event occurred (Table 1); a young adult nearly died during care due to technical difficulty in handling airway equipment and breaks in communication between interprofessional team members.

**Table 1:** Narrative of serious safety event that inspired need for in situ simulation training

“A 28 year old previously healthy male patient was found unconscious in his bedroom by his parents and brought to the emergency department. He was immediately put in the emergency room and interprofessional care team was activated. Two nurses, three nurse technicians, a pediatric emergency physician, a surgeon and an emergency physician arrived within minutes. Nurses attempted first an IV access. Physicians noticed patient was not breathing, so they directed efforts to try to ventilate patient. There were lots of fluids in the airway, so aspiration was attempted, but the aspiration machine did not work properly. The Emergency physician decided to intubate the patient and ordered neuromuscular blocking medication. Attempts to intubate by both the Emergency Physician and Surgeon were not successful, and there was difficulty ventilating the patient with bag and mask. A laryngeal tube was placed, which achieved ventilation. While the care team attempted to adjust transport ventilator parameters, the laryngeal tube cuff ruptured, and there was not another tube available. Since bag-mask ventilation was not effective the Surgeon prepared for a surgical airway. While equipment was being set up the Pediatric Emergency Physician successfully intubated the patient using Glidescope, although the guide wire was also not found. During the acute care for this patient, no leader was identified and many breaks in communication occurred. The patient was transferred to ICU and eventually recovered, but had massive aspiration pneumonia.”	

In this hybrid training program, in situ simulation is combined with just-in-time and just-in-place task training in an emergency department. To our knowledge, this is the first study to combine these simulation-based techniques in a single training of adult and pediatric emergency department providers.

In this study, we aimed to detect different types of LST in this high-risk unit. We attempted this by using a debriefing checklist of in situ simulation scenarios. Secondary objectives were to compare rates of LST detection per team leader profession, shift schedules, and number of participants in the scenarios. We hypothesized that a simulation-based training in actual care areas allows the detection of at least one LST per simulation scenario.

## Methods

This prospective observational study assessed the first year of training from April 2015 to March 2016.

The training was conducted at the emergency bay and at one of the observation rooms of the Ibirapuera Advanced Unit of Hospital Israelita Albert Einstein in Sao Paulo, Brazil. This freestanding emergency department is physically separated by 7 km from our main hospital. It is a private secondary service, working non-stop with urgent and emergency care of adults and children. In 2013, it managed 64,891 patient consults, or an average of 5400 per month [11]. Before this training, a single in situ simulation scenario was conducted in 2013, resulting in the detection of four LST [12].

Analysis of the serious safety event described in Table 1 prompted establishment of interprofessional training. Since our facility is distant from the main hospital and the simulation center, it was difficult to schedule trainings for interprofessional teams; thus, the choice of in situ as the training strategy. Additionally, one of the study authors (TBC) had worked as visiting researcher at Cincinnati Children’s Hospital Medical Center, where he had contact with a well-established in situ simulation program [6]. During night shifts, staff was reduced and less experienced professionals were usually working. Management and senior providers thought this was a more vulnerable time, so it was decided to distribute trainings during all work shifts. Initial planning was to have weekly unexpected simulations [13], but logistical constrains of having to transport simulators and concerns with acceptability led to concentrating scenarios in this simulation week format.

The study population was 135 health care providers, including physicians, nurses, and nurse technicians working at the unit during the study period who could potentially be part of the teams caring for emergency cases. Even though the different work shifts were included in the training, simulations were scheduled independently of any individual professional’s work schedules. Therefore, the selection of participants for each scenario was by convenience. Our goal was to reach at least 80% of the providers during training (108 participants).

The training involved in situ simulation scenarios, in which a simulator was treated at an emergency bay by interprofessional teams working on that shift, followed by debriefing combined with stations of just-in-time and just-in-place self-directed task trainings. Training objectives were to increase safety awareness by detecting LST, to improve management of critically ill patients admitted to the emergency bays, and to reinforce crisis resource management (CRM) principles. In short, ultimately, the goal was to foster patient safety in the emergency department.

Four themes were defined as critical in our needs assessment, which was conducted by consulting the management and senior staff of the hospital and analyzing previous serious safety events at our institution’s emergency departments.

Three scenarios (pediatric, adult, and adolescent patients) were constructed for each theme and alternately repeated during simulation weeks, which occurred every 3 months (Table 2). All scenarios had technical objectives; the management of a specific emergency and objectives based on Gaba’s CRM key points [14].

**Table 2:** Themes and scenarios for each simulation week

Week theme scenario	Arrhythmia		Respiratory insufficiency		Shock		Cardiopulmonary resuscitation	
	Technical	CRM	Technical	CRM	Technical	CRM	Technical	CRM
Pediatric	Infant supra ventricular tachycardia	Prevent and manage fixation errors	Rapid intubation sequence	Cross (double) check	Infant sepsis	Use cognitive aids	Symptomatic bradycardia	Know the environment
Adolescent	Unstable ventricular tachycardia	Re-evaluate repeatedly	Severe asthma exacerbation	Exercise leadership and followership	Anaphylaxis	Use all available information	Ventricular fibrillation arrest	Use good teamwork
Adult	Symptomatic bradycardia	Mobilize all available resources	Respiratory arrest	Anticipate and plan	Adult Sepsis	Communicate effectively	Pulseless electric activity arrest	Allocate attention wisely
Self-directed task training	• Defibrillation • Cardioversion • Transcutaneous pacemaker		• Intubation • Laryngeal mask airway • Transport adult and infant ventilator		• Intraosseous • Ultrasound guided central line • Infant and adult urinary catheter		• Adult and infant CPR • Defibrillation • Pads with feedback	

The patients in these scenarios could be treated by clinical or pediatric teams. We planned to conduct 14 scenarios in weeks 1 and 2, and 15 in weeks 3 and 4, totaling to 58 scenarios. All scenarios utilized simulators (SimMan 3G, SimBaby or SimNewB, Laerdal) and actual drugs and equipment available at the unit were used. Scenarios were scheduled at fixed hours, at times of few anticipated consults. They lasted for 10 min and were immediately followed by 10 more minutes of co-debriefing. Debriefing time was shorter than it would be at the simulation center since the emergency bay had to be ready for patients and participants were on regular work hours and expected to resume patient care immediately after scenarios. The co-debriefing was conducted by two facilitators (a physician and a nurse) that were part of the unit staff but were not on clinical hours. The following clear cancelation criteria were established to not risk patient care: medical supervisor decision, patient at the emergency bays or less than three available professionals. Based on literature, we expected a cancelation rate around 25% [4].

In the week prior to each simulation week, professionals received by email study materials related to the weekly theme, with institutional protocols and key publications. During simulation weeks, an observation room was converted into a training station, with task-trainers and step-by-step guides to procedural skills related to the theme chosen (Table 2). The professionals were encouraged to practice these skills in a self-directed manner.

Categories of LST were measured by analyzing the debriefing checklist filled out by the two facilitators after each scenario. The debriefing checklist was modeled after a similar instrument used by Patterson et al. [4], adapted to the Portuguese language (Additional file 1). Both facilitators completed the checklist during the scenario and the debriefing, and immediately after debriefing they met to compare notes and deliver a final joint version, which was analyzed by the study team.

Since the emergency department treated adult and pediatric patients, team leaders could be emergency physicians or pediatric emergency physicians. There was no fixed team compositions pre-established and scenarios occurred at different hours. Information on team leader, participants and time of simulation was collected to allow comparisons of LST detected per team leader profession, shift schedules, and number of participants in the scenarios.

The Hospital Israelita Albert Einstein’s ethics review board approved the study design, with approval number CAAE 54071816.8.0000.0071.

### Statistical analysis

Categorical variables were described by absolute and relative frequencies; and numerical variables, by means and standard deviations, if they follow a normal distribution, or by medians and interquartile, if otherwise. Numerical variables also included minimum and maximum values. Since it did not present in a normal distribution, the number of LST was compared between groups using Mann-Whitney and Kruskal-Wallis tests.

The software package R version 3.2.2 (R Core Team, 2015) was used. The significance level adopted was 5%.

## Results

**Fig. 1:**
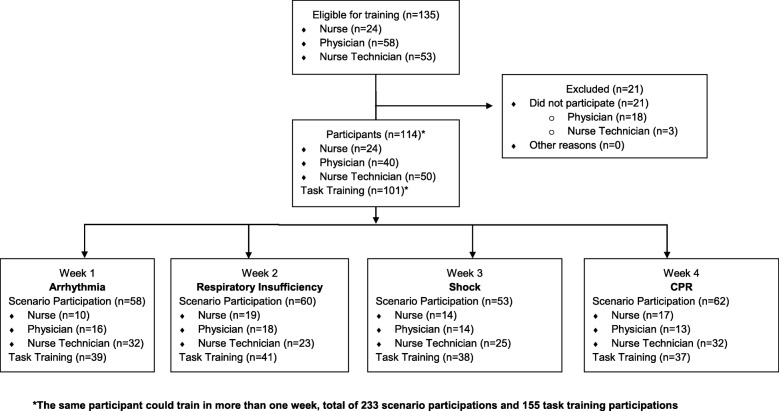
Participant flow diagram

One-hundred and fourteen professionals (84% of the population) participated in this study. Most participants were nurse technicians, which would be the Brazilian equivalent to a licensed practical nurse, representing 43.9% of the participants. The presence in self-training was verified, in its own worksheet, with 101 participants. Figure 1 (participant flow diagram) details characteristics of participants.

The number of scenario cancelations was nine, making the final total number of scenarios to 49 of the 58 proposed. The number of professionals in each scenario ranged from 3 to 36 participants, with a median of 11 (7.0; 13.0). This number included all debriefing participants, which included observers and not necessarily all professionals that treated the simulator. There was no correlation between the number of participants and detected LST, with a correlation coefficient of − 0.08 (*p* = 0.602). The number of scenarios that each professional participated ranged from 1 to 32, with a median of 3 (2.0, 5.25). Fifty-six LST were observed in 49 scenarios, with an average of 1.1 LST per scenario. There was no significant difference in the distribution of LST between categories of team leaders, themes, and shifts (Table 3).

**Table 3:** Latent safety threats per scenario theme, shift, and team leader

	Scenario n (%)	LST median (first and third quartiles)
Total	49	1 [1; 2]
Theme		
Arrhythmia	13 (26.5)	2 [0; 2]
Respiratory insufficiency	12 (24.5)	1 [0; 2]
Shock	11 (22.4)	0 [0; 1]
Cardiopulmonary resuscitation	13 (26.5)	1 [0; 1]
*p* value		*0.563*
Shift		
Morning	20 (40.8)	1 [0; 2.25]
Afternoon	14 (28.6)	0 [0; 1]
Night	15 (30.6)	1 [0; 1.25]
*p* value		*0.397*
Leader		
Emergency physician	24 (49)	1 [0; 2]
Pediatric emergency physician	25 (51)	1 [0; 2]
*p* value		*0.966*

Description by *n* (percentage) and median [first and third quartiles]. Mann-Whitney and Kruskal-Wallis tests

The LST were divided into four categories, namely, medication (e.g., dilution errors, administration, and dosing) with 11 (19.6% of total) LST detected, equipment (e.g., difficulties with defibrillator, ventilator, and intraosseous access) with 23 (41.1%) LST detected, teamwork (e.g., absence of the leader, miscommunication, and unclear division of roles and responsibilities) with 12 (21.4%) LST detected, and others (e.g., laboratory-, personnel-, and radiology-related matters) with 10 (17.9%) LST detected. There was a higher proportion in equipment-related LST, with 41.1% of the total number of LST (*p* < 0.01). Table 4 shows LST by category.

**Table 4:** Latent safety threats by category

Category (*n* = 56)	Theme	Specific threat identified
Medication (*n* = 11)	Arrhythmia (*n* = 4)	No crosschecking of sedative dose
		No check back of verbal order of medication
		No sedation given with transcutaneous pacemaker
		Atropine not given for bradycardia
	Respiratory (*n* = 2)	Information on drug doses not immediately available
		Incompatible drugs in same IV access
	Shock (*n* = 3)	Dilution of antibiotic in high volume
		Delay in preparation of vasoactive drug
		Wrong dose of sedative
	CPR (*n* = 2)	No flush given after epinephrine
		Error in pediatric medication dilution
Equipment (*n* = 23)	Arrhythmia (*n* = 5)	Personal protective equipment not used
		Difficulty adjusting defibrillator
		Lack of familiarity with emergency equipment
		Delay in EKG
		Lack of familiarity with pacemaker pads
	Respiratory (*n* = 8)	Inverted non-invasive ventilation mask
		Wrong size of bag valve mask chosen for child
		Laryngoscope with weak batteries
		Wrong guide wire chosen
		Delay in locating difficult airway bag
		Misuse of transport ventilator
		Protective glasses not used for intubation
		Air leak with transport ventilator
	Shock (*n* = 5)	Ultrasound not available for central line
		Need to anticipate use of emergency equipment
		Inadequate use of intraosseous needle
		Delay in locating intraosseous drill
		Pediatric stethoscope not available
	CPR (*n* = 5)	Capnography not available
		Laryngoscope did not work
		Bag valve mask not connect to oxygen source
		Delay in defibrillation
		Defibrillation pads position inadequate
Teamwork (*n* = 12)	Arrhythmia (*n* = 5)	Clear roles and responsibilities not assigned
		Lack of closed looped communication
		Leader not assigned
		High workload for nurse technician
		Nurse did not call out medication given
	Respiratory (*n* = 2)	Excess number of members of resuscitation team
		Poor workload distribution
	Shock (*n* = 2)	Lack of members in resuscitation team
		Delay of arrival of physician
	CPR (*n* = 3)	No change in compressor
		No person assigned for time keeping
		Incorrect position of resuscitation team
Other (*n =* 10)	Arrhythmia (*n* = 2)	No blood pressure measurement
		Need to standardize oxygen device in pediatric emergencies (catheter vs. non-rebreathing mask)
	Respiratory (*n* = 4)	Delay in requesting lab results
		Delay in monitoring patient
		Allergies not checked
		Delay in intubation
	Shock (*n* = 1)	Poor communication with patient
	CPR (*n* = 3)	Frequent compression interruptions
		Poor compression quality
		Pulse not checked after change in rhythm

## Discussion

This study assessed the effect of combining in situ simulation scenarios with just-in-time and just-in-place self-directed training in an emergency department on detecting LST. The results are consistent with a previous study on in situ simulations at a pediatric emergency department [4] and review articles indicating that simulation is an effective way of detecting LST [15–17].

The rate of LST detection was slightly higher than expected (1.1 per scenario), with a higher proportion of equipment-related than medication and teamwork-related LST. There was no specific theme related to a higher detection of LST. Detecting LST also did not differ among shift schedules and team leader specialties. Some of the equipment needed in the scenarios, such as intraosseous needles [18] and video laryngoscope [19], are often not used by all emergency physicians. New types of equipment, such as pads with resuscitation feedback and a new pediatric ventilator, were also introduced to the unit closely to training, which might explain the higher proportion of equipment-related LST.

LST detected during training were communicated to the emergency department management, which in turn made efforts to mitigate LST found. Part of the strategy to respond to these threats was training-related, mitigated by availability of task trainers and equipment for procedural training during simulation weeks. This was especially important since many LST were related to unfamiliarity with equipment and difficulties in technical procedures. Pharmacists and pharmacist assistants were also included in in situ training after the first week, since medication issues were also often found. Some equipment was acquired in response to threats found, like ultrasound to assist central line catheterization. Other LST were related to system and organizational issues, and changes were made, like changing location of essential materials to ease access, acquiring a white board to annotate verbal orders in emergency situations, and making cognitive aids like table with doses of pediatric medications available. One major change in the emergency room dynamic was also adopted, with fixed emergency teams with pre-defined roles composed of six to eight professionals, to avoid overcrowding or lack of professionals in emergency situations.

This study has limitations. First, the single-center nature of this study makes the results not generalizable. Second, our main outcome, the detection of LST is only a Kirkpatrick level 3 outcome, with safety behavior being assessed during training which could lead to changes in the clinical environment. Although efforts were made to address LST found, we did not establish a formal way of measuring strategies to mitigate any LST found in the study. More important than the location of a simulation scenario is what this activity is trying to accomplish, as often healthcare simulation, and in situ simulation in particular, include a range of behaviors required for safe and effective clinical practice, which might dilute efforts and make it difficult to assess improvement [20]. The in situ program was considered a great diagnostic tool, but it lacked measurement of level 4 outcomes, which could show training-related improvement from clinical outcomes [21]. Another limitation was the short debriefing time, which might have prevented in-depth discussion of some of the LST detected. We classified our LST according to pre-established criteria, which might not describe them as well as a less strict categorization. We relied on our facilitator’s perception of LST detected during scenarios and debriefing and did not use video review to assess LST, which might have permitted a better understating of the nature of LST [22].

Although it was not anticipated as an outcome of this study, the number of days between serious safety events in our institution’s emergency departments has improved from every 122 days to the current rate of once every 365 days since the beginning of our in situ program. We cannot establish a clear causal relationship between training and this outcome, but this may have been a contributing factor to the improvement.

This first year of in situ simulation was considered very successful, initiating a culture change in our emergency department, breaking hierarchies, and allowing a better perception of safety issues. The in situ simulation program has been expanded since it now comprises five different emergency departments within our hospital system. Based on the results of this study, we changed the task training from exclusive self-directed to both self-training and instructor-led training within each simulation week. We also established a formal link between the simulation center and quality and patient safety departments to address the LST discovered during training. We intend to study this expanded program in the future, including measuring level 4 outcomes, such as reduction of serious safety events.

## Conclusions

A simulation-based training program, which combined thematic weeks of scheduled in situ simulation scenarios with just-in-time and just-in place task training in an emergency department in Brazil, allowed a high rate of detecting LST regardless of the training theme.

## Additional file

Additional file 1: In situ checklist (DOCX 151 kb)

## Abbreviations

CPR: Cardiopulmonary resuscitation
CRM: Crisis Resource Management
LST: Latent safety threats
