# Supplementary material for: Detecting latent safety threats in an interprofessional training that combines in situ simulation with task training in an emergency department
Source: Adv Simul (Lond). 2018 Nov 23;3:23. doi: 10.1186/s41077-018-0083-4 (PMC6260660; doi:10.1186/s41077-018-0083-4)
Supplement: Supplementary file 1 — In situ checklist (DOCX 151 kb) [file 41077_2018_83_MOESM1_ESM.docx]

**In Situ Checklist**

| **Scenario** |  | | | |
| --- | --- | --- | --- | --- |
| **Performance and Teamwork** | **Information Shared** | | **Source of information** | |
| (+)  Positive feedback on performance from participants |  | |  | |
| (∆)  Negative feedback on performance from participants |  | |  | |
| Teamwork concepts observed/discussed |  | |  | |
| Additional observations |  | |  | |
| **Latent Safety Threats** | **Information Shared** | **Source of information** | | **Suggested Solutions** |
| Medication |  |  | |  |
| Equipment |  |  | |  |
| Resources (labs, staff, radiology, etc.) |  |  | |  |
| Other |  |  | |  |
